# The impact of conditional cash transfers for HIV prevention on peer relationships: perspectives from female recipients and non-recipients in HPTN 068

**DOI:** 10.1186/s12889-022-14529-3

**Published:** 2022-11-30

**Authors:** Makhosazane Nomhle Ndimande-Khoza, Fiona Scorgie, Sinead Delany-Moretlwe, Amanda Selin, Rhian Twine, Kathleen Kahn, Audrey Pettifor, Catherine MacPhail

**Affiliations:** 1 Wits RHI, Faculty of Health Sciences, University of the Witwatersrand, Johannesburg, South Africa; 2 Carolina Population Center, University of North Carolina at Chapel Hill, Chapel Hill, North Carolina USA; 3 MRC/Wits Rural Public Health and Health Transitions Research Unit (Agincourt), School of Public Health, Faculty of Health Sciences, University of the Witwatersrand, Johannesburg, South Africa; 4 Department of Epidemiology, University of North Carolina, Gillings School of Global Public Health, Chapel Hill, North Carolina USA; 5 School of Health and Society, University of Wollongong, Wollongong, New South Wales Australia

**Keywords:** Cash transfers, Adolescent girls and young women, HIV, Peer relationships, Africa

## Abstract

CCTs are currently being explored for HIV prevention among adolescent girls and young women (AGYW) in Southern Africa. However, little is known about how CCT geared towards adolescents’ influence peer relationships, despite evidence that peer relationships form a critical part of development in adolescence. This article presents findings from a qualitative study that explored CCT recipients’ and non-recipients’ perspectives on the impact of CCTs paid to AGYW on peer relationships.

HPTN 068 was a randomised controlled trial that assessed whether providing CCT to AGYW and their households reduces AGYW’s risk of acquiring HIV. As part of this trial, we conducted interviews and focus group discussions with sub-samples of AGYW (*n* = 39), who were both cash recipients and non-recipients. Through content analysis, we explored ways in which the CCT positively or negatively impacted on peer relationships.

From the recipients’ viewpoint, the CCT improved their social standing within their peer groups. It facilitated peer identity and promoted social connectedness among AGYW receiving the CCT. Receipt of the CCT enabled AGYW to resemble and behave like their peers who had money, allowing their poverty to become “invisible”. The CCT facilitated social interactions, information sharing, and instrumental social support among AGYW. CCT recipients experienced an increase in their social capital, evident in their ability to network, share, and reciprocate with others. However, the CCT also evoked negative emotions such as jealousy, anxiety, and resentment among non-recipients and led to a deterioration of personal relationships.

CCTs have enormous benefits for AGYW, but they may also have a negative impact on peer relationships. The implementation of HIV prevention interventions focused on structural drivers needs to be conscious of these dynamics and ensure that the negative consequences do not outweigh benefits.

## Introduction

In low- and middle-income countries, cash transfers (CTs) have been paid to vulnerable households to provide a social safety net to cover the costs of basic necessities such as food, health care or education. Cash payments have been successful in reducing poverty and improving health and social outcomes [1, 2]. The mechanisms through which CTs have been found to reduce poverty are by improving food security, offering economic autonomy and self-sufficiency, and strengthening households by increasing resilience, enhancing human capital, and consequently reducing intergenerational cycles of poverty [3–6].

CTs have generally been paid to female caregivers rather than male household members based on the evidence that cash controlled by women is likely to benefit the entire household, particularly children [7]. Evaluations of these programmes have linked CTs to increased child immunisation [8] and school enrolment and attendance [9, 10]. Recently, in sub-Saharan Africa, trials assessing CTs for HIV prevention have experimented giving the cash directly to adolescents [11–13]. The trials have either paid CTs solely to the adolescent [13] or paid a portion to the adolescent and a portion to the parent or guardian [11, 12].

Studies examining the effect of CTs on HIV outcomes have documented mixed results. Most studies show that there are benefits to both conditional and unconditional CTs for adolescent girls and young women (AGYW). The Zomba trial in Malawi paid AGYW up to USD 5 and their parents up to USD 10 monthly to assess the effect on HIV prevalence among AGYW [11]. Findings showed that AGYW who received CTs had lower HIV prevalence. They were less likely to engage in risky sexual behaviour and less likely to have to teenage pregnancies and early marriage [11]. In South Africa, HPTN 068, a randomised controlled trial (RCT) assessing the effect of a CT conditional on school attendance on HIV incidence among AGYW, paid USD 10 to AGYW and USD 20 monthly to their caregivers. In this study, adolescent recipients were less likely to report risky sexual behaviour and intimate partner violence than were non-recipients. However, no impact was found on HIV incidence [12]. Qualitative studies forming part of these trials show that CT payment to adolescents increased their autonomy, peer status, self-esteem, and decision-making abilities [14, 15].

Evaluation studies suggest that the payment of CTs to individuals in vulnerable households also has a broader unintended impact on social relationships [16–18]. Research carried out on national social grant programmes in Eastern and Southern Africa has shown that in addition to reducing poverty, the grants also strengthened social networks and promoted participation in community events and social cohesion [18–21]. In Kenya, the CT provision to female caregivers encouraged sharing, borrowing, eating together, and an increased ability to partake in community events [22]. However, these studies also showed that targeting specific households and excluding others can have divisive consequences in close-knit, poverty-stricken communities [23, 24]. Such targeting potentially leads to tension, jealousy, and conflict [16, 25]. Even the eligibility criteria used in CT programmes to identify recipients could threaten social ties [26]. Targeting specific individuals or households and excluding others may induce stigma and resentment between recipients and non-recipients [16, 25, 27, 28].

These findings are important to bear in mind when considering benefits and drawbacks to the payment of CTs to adolescents. Adolescence is a unique developmental stage in which peer relationships play a significant role in one’s development and well-being [29]. A sense of belonging is essential among adolescents, demonstrated by their heightened need for acceptance and social inclusion, and the desire to identify with and participate in events with their peers [30]. Adolescents spend time together, discussing issues such as sex, alcohol, fashion, and hairstyles, and a significant component to peer acceptance is looking and dressing well [31]. The decisions, behaviour and values of individual adolescents tend to be heavily influenced by peer group norms, for example, choosing the same clothes and hairstyles as their friends through a variety of mechanisms, including persuasion, information exchange, modelling, and social interactions [32]. In this way, peers contribute to adolescents’ well-being and perception of themselves and have the potential to become a primary source of support [33, 34].

This background raises several questions about cash payments directed to adolescents. What does the provision of CTs to a sub-group of adolescents mean for peer relations within the larger group? Specifically, what are the unplanned social consequences of cash payments to AGYW on their peer relationships? Answering these questions is critical. There is currently a limited understanding of how direct CT payments to AGYW affect their relationships with peers and others in the broader community. Recent studies that explored the CTs’ influence on adolescent recipients’ interpersonal relationships focused on intra-household relationships [35], and on intimate and platonic relationships with members of the opposite sex [36]. However, a pilot study of a CCT paid directly to AGYW prior to the full implementation of HPTN 068 found that social relationships between young women and their peers remained unchanged. The study showed limited negative impacts on social relationships, such as jealousy between recipients and non-recipients, although the duration of the study may have been too short to observe social impact over time [37]. Furthermore, it was hypothesised that any negative effect might dissolve once people better understood the intervention.

Interventions that seek to improve sexual health outcomes in adolescents should also monitor and assess broader social and inter-personal impacts, which could mitigate or strengthen the impact of these interventions [38]. To contribute to our understanding of the broader effects of CTs directly paid to AGYW on peer relationships and social norms, we analysed qualitative data from the HPTN 068 study (also known as Swa Koteka) in South Africa. This article advances findings from the HPTN 068 pilot study by examining how peer interactions of both female recipients and non-recipients were impacted over a period of three years.

## Methods

### Study setting

The study was located in Agincourt, South Africa, a sub-district of Bushbuckridge, Mpumalanga province, situated near South Africa’s border with Mozambique. This location is characterised by poverty, unemployment, poor infrastructure and temporary local migration, with family members of working age typically migrating to Johannesburg or nearby cities for work and visiting home infrequently [39]. HIV prevalence in this area among those older than 15 years is estimated at 19.4%, with a prevalence of 23.9% among women and 10.6% among men [40].

### The HPTN 068 trial

We report findings from a qualitative study nested within the Swa Koteka trial. Swa Koteka was a Phase III randomised control trial assessing the effect on HIV incidence of CTs conditioned on 80% school attendance among South African young women (*n* = 2448) aged 13–20 years. The trial provided a monthly CCT (R100 ≈ USD 10) to AGYW and their caregivers (parents/guardians) (R200 ≈ USD 20). They received cash deposited into their bank account every month during which they had met the school attendance criteria, for up to 3 years. At enrolment, participants underwent pre-test counselling, sample collection for HIV and herpes simplex virus (HSV)-2 testing, and post-test risk reduction counselling. They also completed an Audio Computer-Assisted Self-Interview (ACASI), a tool used to collect demographics and behavioural quantitative data. Participants had annual study follow-up visits at 12, 24, and 36 months until the study completion date or their planned high-school completion date, whichever came first. Follow up procedures were the same as enrolment [41]. School attendance was collected from local schools monthly. The young women in the control arm of the trial underwent the same procedures, except for receipt of CCT. Social harms, such as experiences of violence due to receipt of CCT or participation in the study, were assessed at each follow-up visit by a counsellor (see Pettifor et al., 2016 for more details).

### Qualitative study design and data collection

A qualitative study was nested within the trial to explore the acceptability of the intervention, the social and relational impact of CTs, and the meanings attached thereto.

### Participant selection and recruitment

From a sample of 2448 AGYW in the trial, we purposively recruited a sub-sample of 39 AGYW for participation in up to six serial in-depth interviews (IDIs) conducted 6-monthly. We used purposive sampling to ensure that sexually active AGYW who reported missing school and those reporting engagement in transactional sex were represented in the sample [35]. Using the trial contact database, potential participants were contacted telephonically and invited to participate in IDIs. A home visit was scheduled where additional information about the study was provided. Participants were asked to provide written informed assent and/or consent. Caregivers provided written informed consent for AGYW who were minors to participate in the qualitative study.

We also randomly selected approximately 120 HPTN 068 CT recipients and non-recipients, for participation in focus group discussions (FGDs). Those who had participated in the IDIs were not eligible to take part in the FGDs. This decision allowed for a larger overall sample and enabled us to capture a more diverse range of perspectives from AGYW in the trial Potential participants were contacted by telephone to invite them to participate in the planned FGDs. Home visits with interested participants were scheduled to obtain written assent and/or consent, as described above.

### Data collection

IDIs and FGDs took place between 2012 and 2015. IDI participants were interviewed twice a year throughout their participation in the trial, providing verbal re-consent at each interview. The IDIs explored perceptions of the CCT programme, use and impact of CCT, exclusion from the programme, and social relationships in households, schools, and communities. All interviews were conducted at participants’ households. The duration of the interviews was 30–60 minutes.

Six FGDs were conducted annually for three years. FGDs explored participants’ thoughts about the CCTs and the impact of the cash on peer relationships among recipients and between recipients and non-recipients. The FGDs were conducted on weekends and during school holidays at local schools. Each FGD comprised 10–12 participants and lasted approximately 60 to 90 minutes.

The IDIs and FGDs were facilitated by four trained interviewers, all of whom were women, local residents, fluent in XiTsonga, and with a minimum Grade 12 education. The study was approved by the Human Research Ethics Committee of the University of the Witwatersrand, the Institutional Review Board of the University of North Carolina, and the Provincial Department of Basic Education in Mpumalanga Province.

### Data analysis

Interviews were audio-recorded and subsequently transcribed and translated into English by the interviewers. Transcripts were checked for quality by one of the authors (MNK) through comparisons with the audio files. The transcripts were subsequently imported into Atlas.ti. Transcripts were coded using a coding frame that was both inductively and deductively generated by two of the authors (CM and MNK), following a framework approach [42]. This approach entailed the generation of themes from *a priori* hypotheses and questions that guided the objectives and aims of the study (deductive), and issues derived from the data (inductive). Coding was conducted by five trained researchers, who were also involved in data collection. Coders sought to attain acceptable intercoder reliability (ICR) scores on early coded transcripts [43]. A random sample of 10% of the transcripts was double-coded throughout the study to ensure that ICR remained acceptable.

## Findings

### Description of participants

A total of 39 AGYW took part in the interviews. Twenty-six AGYW were interviewed three times, 12 interviewed four times, and six interviewed five times. Interview participants comprised 18 CCT recipients and 21 non-recipients, and their age range was 13–20 years. A total of 18 FGDs (*n* = 108) were conducted during the three-year study period. Study participants came from households where the majority were dependent on child support grants, and on income from single parents and migrant remittances. Most households were made up of 5 to 8 family members, with grandmothers mostly assuming the role of guardians if both parents were absent or had died.

### Positive consequences of the CCT on peer relationships

#### Allowing recipients to identify with peers: ‘I am now like other children’

Receipt of the CCT enabled AGYW to fit in within their peer groups in terms of how they looked and ate, and the activities in which they participated. CCT recipients reported that before the CCT trial, there was a distinction between ‘girls from ‘rich’ households’ and ‘girls from ‘poor’ households.’ Young women from better-off families had pocket money to take to school, which afforded them better meals during lunch breaks at school, had proper school uniform and supplies, and had better clothes than those from poor family backgrounds, who relied on ‘free food from the feeding scheme’ and lacked some school necessities. Consequently, young women from poor households felt that they were different from their peers – they ‘felt out of place’. As Yolisa (recipient, IDI) indicated, ‘if I go to school without money, it’s like I’m not like other people.’ This affected some participants emotionally. Thembi (recipient, IDI) recounted that before she received the CCT, she was unwell and dreaded going to school, knowing that her friends would have money and she did not. Emyoli also shared a similar experience:

‘In the past, I was not feeling well; I was feeling like I’m down [depressed] when I’m with my friends, and they have money, and I don’t have it’ (Emyoli, recipient, IDI).

For other participants, these feelings were exacerbated because they were ridiculed by their peers for lacking desirable items. For example, Thandeka (recipient, IDI) reported that girls from well-off households had branded clothing, while she wore the cheapest, unbranded clothes.

“In my class, there are girls who always wear expensive clothes like Nike, Levi’s jeans … I always wear no names from PEP [store] or Mr Price, you see. I don’t feel good.” (Thandeka, recipient, IDI)

She also said that she was hurt by her peers ridiculing her for lacking necessary school supplies.

This was echoed in one FGD where a participant shared experiences of young women who were mocked for compromising their personal care and hygiene because they could not afford to buy cosmetics. In some instances, this led to young women missing school. She recalled,

‘ … some of the kids were not going to school because they were afraid because boys were laughing at them because they smelled bad under their arms’ (recipient, FGD).

Young women reported that receipt of the CCT allowed them to ‘be like other young women’, a phrase used by many participants to describe how the CCT had changed their lives in relation to their peers.

‘When the other young women buy something at school, I also buy because I do have money.’ (Emyoli, recipient, IDI)

‘When you go to school, you feel like other learners like having lunch.’ (Zama, recipient, IDI)

‘Since I take part in the study, I do have money to carry when I go to school every day like other learners.’ (Sbonga, recipient, IDI)

These quotes show how significant it was for young women to feel the same as their peers, particularly when it came to having pocket money at school. Thandeka reported that the CCT had made a difference in her life and that her peers had stopped making fun of her:

‘I have my school bag, school uniform, I feel happy about it. At school they were laughing at me because I was using a shopping bag to carry my books, now they are no longer laughing at me.’ (recipient, IDI)

The cash enabled recipients to fit in and not be seen as different from other young women; this gave them a sense of peer identity and a sense of belonging. Receipt of CCTs enabled AGYW to not stand out as different from their peers, which had previously made them vulnerable to teasing or bullying.

#### Facilitating interactions, conversations, and information sharing among young women

Receipt of the CCT appeared to have created networking opportunities between AGYW. Generally, the participants travelled together to the post office to collect or withdraw the CCT. A young woman in an FGD mentioned that networking with others had improved as a result of participation in the CCT trial:

‘You find that you did not have someone’s phone (contact) number, but you find that now you can communicate and ask one another what is going on. Like the other day, they came home, and then I asked her if they [trial staff] did come to her house as well … but we did not communicate before [the trial].’ (recipient, FGD)

Some non-recipients observed that CCT recipients befriended one another, formed groups, and hung out together:

‘Those who are getting money, they are grouping themselves as they are getting money; because of the money that they are getting from the study…’ (Lettie, non-recipient, IDI)

Londiwe, a non-recipient, also claimed that ‘now they [recipients] get together, but in the past, it was not like that.’ These findings suggest that participation in the CCT trial may have led to new friendships, particularly among recipients.

Unsurprisingly, there were conversations between the girls about the CCT and subsequently, study group allocation became common knowledge. The conversations entailed sharing subjective experiences, perceptions of the trial, advising and guiding each other, and sharing trial information with one another. These took place in communities, schools, during study visits, and at the post office bank when recipients went to withdraw their CCT payments. Conversation among recipients was mostly about the CCT. Lucy, a CCT recipient, remarked, ‘We ask each other if you received it [money]?’

Similarly, Khensani stated,

‘we are chatting about many things like when are you going to take money from Wits [the trial]?’ (Recipient, IDI).

CCT recipients shared their spending plans and advised one another on how to spend the cash appropriately. As a CCT recipient in the FGD indicated,

‘we are able to share how we spend the money, and if she misuses the money, we tell her that it is not good - you have to use it on necessary things.’

CCT recipients also shared complaints and frustrations related to cash payments, such as missed payments or dissatisfaction with the amount of cash received. Nkateko recounted the following about her friend: ‘She is always complaining that they must add money.’ (recipient, IDI).

Thuthu also remarked,

‘They [recipients] are complaining that the amount is too little; it can only buy a bag of mealie meal.’ (non-recipient, IDI)

During an IDI, one participant stated,

‘our relationship is good because we chat with the other girls and we guide each other. We talk about good and bad things and how we should take care of ourselves.’ (Zinhle, recipient, IDI)

#### Facilitating reciprocity and resource sharing

Most CCT recipients spoke of how the money allowed them to assist friends and family members financially, particularly those who gave them money prior the CCT trial. They described how, before the trial, girls from ‘poor’ households had relied on those who had pocket money for lunch at school. Not only did the receipt of the CCT enable recipients to be more self-reliant, it also improved some recipients’ ability to pool finances with their friends and enhanced these relationships by allowing reciprocity. A young woman in a recipient-only FGD remarked,

‘Before I received this money, she [friend] was able to help me, and now that I have my own money, we can help one another (FGD3).

Another participant reported,

‘When I go to school, the money that I get from Swa Koteka, I use it as pocket money. When my friend doesn’t have pocket money, I share food with her, and if I don’t have pocket money, she shares with me.’ (Thoko, recipient, IDI)

The ability to help in this way therefore gave recipients a sense of personal fulfilment, as they could now also return the favour. The ability to reciprocate financially also gave recipients a higher social standing, as access to cash appears to have increased one’s visibility and perceived value in the peer group.

Recipients could also lend money to friends who were non-recipients. Noma, a non-recipient, had a friend who was a CCT recipient. She reflected on the day she found out that she would not be receiving the monthly CCT payment:

‘I just told her that ‘my friend, you know I did not get the money’ and then she just said, ‘do not worry … if you have a problem like you need something, just tell me I will share with you … ’ When she has money, she comes to my class and gives me money and says, ‘go and buy food.’ (Noma, non-recipient, IDI)

Only in a few instances did it seem that the CCT had negatively affected the reciprocity between peers and the culture of lending and sharing. Some participants felt that once CCT payments started, the inclination to share disappeared. Non-recipients observed that some recipients found it tiring to be the only one buying items for friends who never contributed. As one FGD participant explained,

‘You go to the market together, and she buys you snacks for two days, and after that, she will complain that ‘I am not going to buy her stuff anymore because she always does not have money; I cannot, anymore.’ (FGD1, non-recipients)

#### Negative consequences of the CCT on peer relationships

As hinted at above, we found a few instances where CCTs appeared to have negatively impacted on peer relationships. This negative impact took the form of an increase in negative emotions, rumours, teasing, stigma and gossip. Disrupted friendships was a further negative consequence.

#### Negative emotional consequences

Some conversations by non-recipients in the IDIs and FGDs portrayed cash payments to their counterparts as unfavourable – triggering negative emotions such as jealousy, anxiety, hurt, and resentment. Participants said, ‘I become worried’, ‘I feel heartaches’, ‘Sometimes, my heart breaks’, and ‘I get bored [irritated]’. These emotions were often prompted when recipients talked about the CCT. Sindy reported,

‘if we told them [friends not getting cash] that we are going to the post office to collect money, they become angry.’ (Sindy, recipient, IDI).

These negative emotions were also triggered when non-recipients observed recipients’ purchases, such as lunch at school or a new outfit.

‘We do have jealousy because those who are in the intervention [recipients] eat well. They buy cool drinks, fish, and chips; that makes us jealous, as we are in the control arm [non-recipients].’ (Mantwa, non-recipient, IDI).

The non-recipients felt that the recipients ‘always spoke about the Swa Koteka money’. Some non-recipients interpreted this as being boastful. In one of the FGDs, a non-recipient reported,

‘They [recipients] are boasting on us saying ‘on Friday we are getting our payment, I am going to buy chocolate…”

These encounters made non-recipients jealous and feel left out since they had neither the money nor the items bought with it by recipients. One young woman in the FGD narrated the following example:

‘You find that you are three friends and then those two got the [CCT] payment [intervention arm] and you are in the control [arm] and then find that on Saturday it’s the 1st [date], and they are going to get the money, and then one says ‘my friend, let’s go to the post office and withdraw money because I saw a jersey at Fashion World [store],’ and find that you [a non-recipient] sat alone and you do not have anything (money) and in that way, you feel like they are discriminating [against] you.’

In an FGD, one young woman described a scenario that triggered negative emotions:

‘Let us say we are friends and when we are hanging out, and then P11 [another FGD participant] says “tomorrow I am going to Thulamahashe [shopping centre], I am going to get the money.” Definitely, that will hurt me. She is going to get the money, and I do not have the money or money to carry to school, and she is telling me that she is going to withdraw the money and her family is wealthy, they have everything, she wears Carvella [a luxury brand of shoes], and I am wearing cheap takkies [trainers/gym shoes].’

Hearing recipients talk about their money, and the fact that recipients’ lifestyles had visibly improved meant that spending time with recipients was distressing for the non-recipients. Many of these non-recipients did not explicitly express or show their anxiety; instead, they tried to hide it:

‘When I am with them, my heart breaks, but I do not show them; when I am alone, I can see that it is not fair.’ (Mbali, non-recipient, IDI)

However, these negative emotions were also sensed by some recipients, even if non-recipients were discreet in showing it.

‘When I buy something now, those who are not getting the money… they get angry because she knows that she does not have money and, at that time, she hates you (FGD, recipient).’

Thandeka, a cash recipient, also alluded to this:

‘It was difficult when you talk with them [non-recipients]. You can feel that this person is not happy, especially if you can tell her that tomorrow you are going to the post office to withdraw money.’ (Thandeka, recipient, IDI)

In some instances, recipients reported that they stopped talking about the CCT money altogether to avoid upsetting the non-recipients.

#### Rumours, teasing, gossip, and stigma

Despite ongoing community engagement and information activities, participants noted that most of the girls who were not part of the study did not have accurate information about the CCT trial. As a result, several rumours about the study circulated about who was being offered or not offered the CCT. Some of the rumours were overlapping and contradictory. On the one hand, rumours circulated that cash was being given to AGYW who tested positive for HIV. The assumption was that the CCT was to enable them to buy healthy food to manage their HIV. Some believed that non-recipients enrolled in the trial were not being given CCTs because they were HIV positive and had ‘unclean blood’. On the other hand, there was another rumour that cash was being paid to girls who were HIV negative as they had ‘clean blood.’ Others believed the CCT was only being given to AGYW from poor households.

Another rumour reported by participants emerged around the fact that one of the study procedures necessitated the drawing of blood to run various tests. For some people not enrolled in the study, blood draws meant that CCTs were payments for selling or donating blood.

‘They say they are withdrawing our blood and sell it. They are making fun of us that we are selling our blood.’ (Mpumi, recipient, IDI)

Non-recipients were teased for ‘giving their blood for free.’ They were also teased for choosing a ‘non-winning ticket’ [randomisation into the control study arm]. Mantwa, a non-recipient, recalled, ‘they say we are not clever because we chose to [remain] in the control [non-benefitting] arm.’

While much of this teasing, jealousy, rumours, and stigma apparently emanated from young women outside the CCT trial, teasing also occurred between recipients and non-recipients enrolled in the trial, despite them understanding the randomisation process underpinning the allocation of participants to the two study arms.

Recipients were also teased about the amount of cash they received. One recalled,

‘They say you think you are smart; meanwhile, you only get R100, and R100 is nothing to us because it is a small amount.’ (Yandi, recipient, IDI)

Among the non-recipients in the trial, jealousy was also tied to the belief that some CCT recipients were undeserving. Non-recipients highlighted instances where they felt CCTs were being paid to young women from well-off families and that impoverished families had been left out.

‘We feel the pain as our family background is bad, and when you look at those who got the money, they do not care; that is why they don’t have something important to do with this money because they are coming from rich families.’ (YW FGD, non-recipient only)

The non-recipients suggested that the targeting criteria for CCT recipients should include a socio-economic background assessment and means-testing. They believed that CCT should be given to only low-income or poor households.

When you do it [the CCT trial] again, they do not make us choose [randomise]. The people who go around in our households [census] have to look for the situations, like, in this household there is a big house that is roofed by tiles, so it means they eat well … [but] here there are three rooms so definitely there is no way they could eat well. And then when you sit down and check those forms you will see that ok, at P10’s household they are poor, so we have to give her intervention [CCT].

Besides one exceptional incident of physical assault, in general, the girls managed the teasing and stigma by considering it harmless and laughing it off or by suppressing their feelings.

#### Disrupted friendships

Significantly, a few AGYW recipients reported ending friendships with friends (non-recipients). In the one instance, Sasa (recipient, IDI) explained that before the trial, she and her friends had influenced each other to miss school, but since she started receiving the CCT she no longer missed school out of fear that she would not receive her school-conditioned cash payment. As a result, she grew apart from her friends. In another instance, a participant reported,

‘Yes, our relationship changed, since I get money, we no longer spend time together. Before [the trial] we were bunking school and going to taverns, but now I decided to focus on my schoolwork.’ (Musa, recipient, IDI)

Overall, however, most of the participants reported that friendships did not change during the course of the trial. For some, the CCT only disrupted peer relationships at the beginning of the trial but these settled over time, as the trial progressed. Senzi, a non-recipient, said, ‘There is no change with my closest friends, like I said, because we grew up together.’

Other participants believed that a change in friendships would be unjustifiable, considering that the CCT was only operational for a limited period. Some participants reported that no changes had occurred because their friends (all enrolled in the study) understood the randomisation process and that receipt of CCT depended entirely on ‘luck.’ It was also evident that many recipients had invested in existing friendships, which they deemed safe and reliable. This investment potentially mitigated the likelihood of relationships with friends deteriorating as a result of the CCT.

## Discussion

Current studies on CTs geared towards AGYW for HIV prevention have primarily focused on education and HIV outcomes, with little attention paid to impacts on peer relationships. Yet CCTs for HIV prevention may have impacts beyond HIV outcomes; they potentially have spill-over implications for other aspects of recipients’ lives. This study contributes to the growing body of research that seeks to understand the broader social implications of CTs for HIV prevention.

Our findings demonstrate that CCTs provided to encourage school attendance with the primary goal to reduce HIV in AGYW also had unintended consequences for peer social relationships. From the recipients’ viewpoints, CCTs improved their social standing within their peer groups, facilitated peer identity, and promoted social connectedness among AGYW receiving the CCTs. Receipt of CCTs enabled AGYW to look and act like their peers who had money, and in so doing, to diminish visible signs of their poverty. This gave young women a sense of belonging derived from conformity to a set of positive – and shared – social norms. CCTs also facilitated social interactions, information sharing and instrumental social support between AGYW. CCT recipients experienced an increased ability to network, share, and reciprocate with others, which in turn potentially increased their social capital. Evidence shows that improved social connectedness among peers is significant. Social interaction, resource exchange practices, and reciprocity form an integral part of adolescent development and social capital generally [44]. Social ties provide a sense of interdependence and belonging, which are often sought after by adolescents and associated with improved personal and social well-being [44, 45].

Paradoxically, for the non-recipients, the CCT trial disrupted peer norms of ‘homogeneity’, with the recipients being resented for seeming to be ‘better’ than their peers who were non-CCT recipients. Giving CCTs to one group of young women and excluding others elicited negative emotions among non-recipients, and triggered some gossip, teasing, and rumours. However, these were slight disruptions overall and did not last long, with most young women reporting that they had maintained relations with their existing friends. Similar findings were observed in our pilot of HPTN 068, that showed that jealousy, teasing, gossip and tension should be anticipated in CCT programmes involving adolescents [35].

Consistent with the literature, our findings illustrate how the psychosocial experiences of poverty were negative for young women. Being different from peers, exclusion, and failure to meet social expectations generated feelings of distress, indignity and humiliation. As a result, poverty and social exclusion could lead to AGYW engaging in negative coping strategies, including engaging in sexual risk behaviours [46, 47]. In this context, CTs paid to vulnerable AGYW may reduce vulnerability by improving their economic well-being and social standing. In the context of HIV prevention, these results are significant as evidence shows that social connectedness to peers has crucial health implications for adolescent development [48, 49]. Strong social connections with helpful peers and sharing of health information can support positive health behaviour and buffer health risk-taking [48–50]. Studies have shown that interventions that build social ties are more likely to influence HIV related outcomes in the long run. However, this impact depends on the content and resources available from social connections [47, 51, 52]. The current study findings underscore the building of social capital among peers as a potential pathway by which CCT could reduce HIV risk, depending on whether these interactions encourage negative or positive behaviour.

Surprisingly, cash recipients made instinctive associations between receipt of CCT and positive/ good behaviours. They shared narratives about shedding bad behaviour since the trial had started and distancing themselves from the friends of ‘bad’ influence; for example, friends who skipped classes or those who hung out in taverns. Recipients reported that instead they focused on their schoolwork and church, and spent the money wisely. However, it is unclear what informed this behaviour as trial organisers did not prescribe how AGYW should use the CCT, aside from encouraging school attendance. These findings suggest the trial and conditionality of the CT, school attendance, as well as other trial procedures such as HIV testing might have led to the CCT being interpreted as promoting ‘good behaviour’ in general. While these findings require further exploration, they highlight another possible way a CCT may reduce risky behaviour among AGYW recipients.

To a lesser extent, our findings suggest that provision of CCT to selected AGYW maintained or widened the gap of socio-economic inequality among peers – AGYW who were lacking before the CCT remained in that position because they were not selected to receive the CCT, yet peers who were thought to be better off or those who were at the same level with them (non-recipients) before the CCT, progressed. These findings indicate that CCT given to certain young women and excluding others could potentially negatively impact on HIV outcomes. In line with evidence on the role of peer pressure on HIV risk behaviours, a desire for respectability, and a desire to appear ‘rich’ among non-recipients has the potential to drive AGYW towards risky sexual behaviours in order to achieve parity with their peers who are CCT recipients. In the HPTN 068 trial, AGYW were allocated to receive CCT through a randomisation process. In a real-life context, some of these issues may be less significant as CTs are generally allocated according to need. However, this is not to say these issues may not come up even in a needs-based approach, as previous studies have reported unhappiness with how decisions are made, and cut-off points and criteria used to define the needy [16, 17]. This may be an issue particularly in communities where most people may be considered poor, despite the criteria used to allocate CTs.

Findings from the current study highlight the critical role peers can play as a support structure for AGYW, and their potential role in both mitigating and fuelling social vulnerabilities associated with HIV risk. These findings have important implications for research and CT programming for HIV prevention that seeks to optimise the impact of CTs’ HIV-related outcomes. Our results point to the need for researchers to recognise existing social connectedness and the opportunity it poses for CT programming targeting AGYW for HIV prevention. They should seek ways to leverage peer interactions and social ties, and use these as platforms to promote sexual health, build critical consciousness, and modify harmful norms to maximise HIV impact. CCT programming targeting AGYW should monitor peer interactions and assess these interactions as a potential pathway CCT reduce HIV risk in AGYW. This could provide valuable evidence given the literature that shows that social connectedness to peers (mutual feelings of trust, reciprocity, shared norms and identity and information and resource sharing) has crucial health implications for adolescence development. Strong social connections with helpful peers and sharing of health information can support positive health behaviour and could buffer health risk-taking [49, 53]. Cross-sectional studies have shown that interventions that build social ties were more likely to influence HIV related outcomes depending on the content and resources available from the social connections [52, 54].

CT programmes targeting AGYW should incorporate peer and community engagement processes such as peer-led strategies and social mobilisation to leverage these social ties. CT programmes should include education that ties together the social and health implications of behaviours and social structures. This may enhance the impact on HIV outcomes, given the evidence that effective HIV prevention interventions also consider social networks which can influence behaviour, communication and norms [55]. These findings are complementary to evidence that underscores the need for multiple combined interventions for HIV prevention [56], and CTs programming provides a platform for this work to occur.

Overall, the idea of paying CCT directly to AGYW seems to have been instrumental in shoring up the positive outcomes of this intervention overall. Adolescent recipients made their own arrangements for collecting the CCT, and devised their own spending plans. There is a reason to believe that if cash was paid to and controlled by caregivers only, it would have been limiting for adolescents. AGYW would not have had a chance to accompany one another to the post office to collect their CCT or engage in activities that allowed interactions between them. The findings indicate how paying CCT directly to AGYW contributes to their personal and social well-being.

One important contribution of this study is that it captured the voices of the non-recipients, which are often missing from studies of CCTs. These voices are significant as they represent the views of the broader community on the impact of CCTs to a specific group and illustrate something of the potential unintended consequences of this intervention. In our study, non-recipients’ concerns were expressed through negative emotions such as jealousy, as well as stigma, and rumours. Considering the evidence that shows the association between social networking, peer pressure, and HIV risk [47, 53], CCT programmes need to find ways to monitor and mitigate the risk. We, therefore, suggest that in poverty-stricken communities, CCTs should be provided to all AGYW in a setting, and not only to a selected few. In South Africa, universal targeting used in social grants has proven to limit unintended adverse effects on community relationships [18]. In instances where CCTs are offered to selected AGYW, the eligibility criteria should be clear, as imprecise and random eligibility criteria could lead to unintended negative consequences [18].

This study was not without limitations. We only gathered perspectives of AGYW who were enrolled in the trial. Including voices of peers outside of the trial would have brought additional insights about the CCT trial and the experience of exclusion. The perspectives of male peers are also missing from this analysis, but are reported elsewhere [36]. Furthermore, the analysis did not assess interactions over time; consequently, it is not possible to say whether interactions continued beyond the timeframe of the intervention. Lastly, while it is evident that some positive impacts resulted from receipt of CCT, it is also possible that some of this positive impact was influence by trial participation than CCT impact; however, positive impact was largely reported by CCT recipients as opposed to non-recipients which suggests CCT contributed to positive impacts.

## Conclusion

This is one of the first studies to explore the social impacts of adolescent directed CCTs. Overall, our findings indicate that CCTs have the potential to enhance the quality of AGYW’s peer relationships. However, they may also induce social changes which have negative consequences. The implementation of HIV prevention interventions focused on structural drivers needs to be conscious of these dynamics and ensure that the negative consequences do not outweigh benefits. Our study confirms the critical role of peer relationships for adolescents’ well-being. This study also echoes research highlighting that a more holistic approach is necessary when assessing the impact of CTs – understanding not only intended outcomes but also the broader social and relational effects of CT programming.

## Data Availability

All audiotapes and transcripts of interviews are stored at Wits Reproductive Health and HIV Institute, South Africa. They are available from the study corresponding author on reasonable request.

## Abbreviations

ACASI: Audio Computer-Assisted Self-Interview
AGYW: Adolescent girls and young women
CT: Cash transfer
CCT: Conditional cash transfers
HPTN: HIV Prevention Trials Network
